# GAD65 autoantibodies and glucose tolerance in offspring born to women with and without type 1 diabetes (The EPICOM study)

**DOI:** 10.1002/edm2.310

**Published:** 2021-11-19

**Authors:** Sine Knorr, Magnus C. Lydolph, Birgitte Bytoft, Zuzana Lohse, Tine D. Clausen, Rikke Beck Jensen, Peter Damm, Kurt Højlund, Dorte M. Jensen, Claus H. Gravholt

**Affiliations:** ^1^ Steno Diabetes Center Aarhus Aarhus University Hospital Aarhus Denmark; ^2^ Department of Endocrinology and Internal Medicine Aarhus University Hospital Aarhus Denmark; ^3^ 4326 Department of Autoimmunology and Biomarkers Statens Serum Institut Copenhagen Denmark; ^4^ Department of Obstetrics Center for Pregnant Women with Diabetes Rigshospitalet Copenhagen Denmark; ^5^ Steno Diabetes Center Odense Odense University Hospital Odense Denmark; ^6^ 60170 Department of Gynaecology and Obstetrics Nordsjaellands Hospital Hilleroed Denmark; ^7^ 53146 Department of Growth and Reproduction Rigshospitalet Copenhagen Denmark; ^8^ Institute of Clinical Medicines Faculty of Health and Medical Sciences University of Copenhagen Copenhagen Denmark; ^9^ Department of Gynecology and Obstetrics Odense University Hospital Odense Denmark

**Keywords:** adolescent, autoimmunity, diabetes mellitus, GAD65 autoantibodies, pregnancy, type 1

## Abstract

The aims of this study were to examine presence of GAD65 autoantibodies (GAD65aab) in offspring born to women with type 1 diabetes (T1D) and controls and if more were GAD65aab‐positive if diagnosed with diabetes or pre‐diabetes. This EPICOM study is a prospective follow‐up study focussing on pregnancies complicated by maternal T1D. The EPICOM study includes offspring (*n* = 278) born to mothers with pre‐gestational T1D between 1993 and 1999 and matched un‐exposed controls (*n* = 303). Age at the time of follow‐up was 16.7 years (13.0–20.4 years). GAD65aab was measured using the Glutamic Acid Decarboxylase Autoantibody RIA kit from RSR^©^. An Oral Glucose Tolerance Test (OGTT) was performed, and abnormal glucose tolerance was defined as having either diabetes, impaired fasting glucose (IFG) or impaired glucose tolerance (IGT). GAD65aab could be measured in 561 participants. Of these, 17 (3%) were positive for GAD65aab (≥25 U/ml) with 11 (4%) offspring being born to women with T1D and 6 (2%) controls. The difference in GAD65aab status was not statistically significant (*p* = .2). One was diagnosed with GAD65aab‐negative diabetes during the study, 18 were diagnosed with IFG, and 44 with IGT. Overall, more were GAD65aab‐positive if diagnosed with abnormal glucose tolerance (*p *= .03). We found no association between GAD65aab status and HOMA‐IR, HOMA‐IS, birthweight, mode of delivery or maternal BMI prior to pregnancy. Our study found no overall difference in GAD65 status between offspring born to women with T1D and their matched controls. However, among the participants diagnosed with pre‐diabetes more were GAD65‐positive.

## Graphical abstract

In this study, we describe how adolescents diagnosed with pre‐diabetes more often are GAD65 autoantibody positive. However, among those diagnosed with pre‐diabetes, we find no evidence of more offspring born to women with type 1 diabetes being GAD65 autoantibody positive than their matched controls. In addition, we find no association between GAD65 autoantibody status and birthweight, mode of delivery, current weight or maternal pre‐pregnancy BMI, but GAD65 autoantibody status is associated with a higher maternal pre‐pregnancy HbA1c.

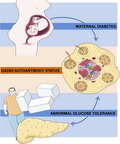

## 1 INTRODUCTION

Intrauterine exposure to maternal hyperglycaemia is associated with a less favourable metabolic profile in the offspring, including an increased risk of developing diabetes and components of the metabolic syndrome.^1^, ^2^, ^3^ This was also the case in the EPICOM study, where adolescent offspring of women with type 1 diabetes (T1D) had an increased risk of pre‐diabetes and higher BMI.^2^ Offspring of mothers with T1D have a higher risk of T1D per se compared with the background population but a lower risk compared with offspring of fathers with T1D.^4^ Since T1D is preceded by a pre‐clinical period displaying autoimmunity, our hypothesis was that GAD65 autoantibodies (GAD65aab) have the potential for identifying offspring born to women with T1D at risk of developing diabetes or pre‐diabetes.^5^ The appearance of GAD65aab has been found to be relatively stable over years and occurs primarily from preschool years up until the teenage years.^6^ Thus, our aims were to examine the presence of GAD65aab in offspring born to women with T1D and controls and if more were GAD65aab‐positive if diagnosed with diabetes or pre‐diabetes.

## 2 MATERIALS AND METHODS

This present study is a sub‐study (secondary analysis) of the EPICOM (EPIgenetic, genetic and environmental effects on growth, COgnitive functions and Metabolism in offspring of women with type 1 diabetes) study, a prospective nationwide follow‐up study including offspring born to women with pre‐gestational T1D between 1993 and 1999 (*n* = 278) and matched controls (*n* = 303). The study has been described previously.^2^ Only singletons and the first child per mother during the study period were included in the EPICOM cohort. The controls were matched according to sex, date of birth and postal code as a marker of socioeconomic status. The participants were studied after an overnight fast, and the clinical examination was carried out between April 2012 until October 2013 at three hospitals in Denmark (Aarhus, Copenhagen and Odense). The protocol is in accordance with the Helsinki Declaration and approved by the regional ethical committee (M‐20110239). Informed consent was obtained from all participants or their parents. Clinical Trial Registration number: NCT01559181.

GAD65aab was measured using the Glutamic Acid Decarboxylase Autoantibody RIA kit from RSR^©^ and was defined as positive if GAD65aab ≥25 u/ml (WHO‐units).

We performed a standard 2‐h Oral Glucose Tolerance Test (OGTT) by using a glucose load of 1.75 g/kg body weight up to a total of 75 g after an overnight fast. Abnormal glucose tolerance was defined as being diagnosed with either diabetes, impaired fasting glucose (IFG) or impaired glucose tolerance (IGT) during the study day using WHO 1999 diagnostic criteria.^7^ Insulin sensitivity was evaluated by HOMA‐IR and insulin secretion by HOMA of insulin secretion (HOMA‐β).^8^, ^9^ Offspring birthweight was calculated as z‐scores (according to sex and gestational age). Height and weight standard deviation scores (SDS) were calculated using a normal Danish reference material.^10^ Body mass index (BMI) was calculated as weight in kilograms divided by height in metres squared, and BMI‐SDS was calculated using the normal Danish reference curves.^10^ Continuous variables with symmetric distribution are presented as means and standard deviation (SD), continuous variable with skewed distribution as medians and interquartile range (IQR) or range. Comparison of groups was performed using Student's t test, Wilcoxon rank‐sum test, Chi2 or Fisher's exact test. Statistical analyses were done in STATA 13.1.

## 3 RESULTS

After excluding offspring with diabetes diagnosed prior to the study (*n* = 1), material was accessible for GAD65aab measurement in a total of 561 participants from the EPICOM study ((T1D offspring *n* = 267 and controls *n* = 294) Table 1). Of these, 3% (17/561) were positive for GAD65aab with 4% (95% CI 2–7) (11/267) among offspring being born to women with T1D and 2% (95% CI 1–4) (6/294) among controls. The difference in GAD65aab status was not statistically significant between the two groups (*p *= .2).

**TABLE 1:** Baseline and follow‐up characteristics of adolescent offspring born to women with type 1 diabetes and matched controls, divided according to GAD65 autoantibody status

	GAD65aab‐positive *n* = 17	GAD65aab‐negative *n* = 544	*p*‐value
Follow‐up data			
Exposed to type 1 diabetes, *n* (%)	11 (65)	256 (47)	.2^†^
Age (years)	17.1 (1.5)	16.7 (1.7)	.5
Male sex, *n* (%)	7 (41)	219 (40)	.9^†^
Weight (kg)	65.4 (12.9)	63.6 (13.0)	.9
Weight SDS	0.2 (1.3)	0.4 (1.2)	.5
Height (cm)	171 (10.5)	171 (9.0)	1.0
Height SDS	−0.2 (1.2)	0.1 (1.1)	.4
BMI	21.2 (19.7–23.0)	21.7 (19.8–24.2)	.9
BMI (SDS)	0.3 (1.3)	0.5 (1.2)	.6
Abnormal glucose tolerance % (+/‐)	42 (5/12)	12 (58/486)	.03^‡^
Diabetes % (+/‐)	0 (0/17)	0.2 (1/543)	1.0^‡^
IFG % (+/‐)	13 (2/15)	3 (16/528)	.1^‡^
IGT % (+/‐)	21 (3/14)	8 (41/503)	.1^‡^
HbA_1c_, mmol/mol [%]	34 (30–38) [5.3 (5.2–6.4)]	33 (22–45) [5.2 (4.2–7.4)]	.7
HOMA‐IR	2.0 (1.6–2.8)	2.1 (1.6–2.8)	.9^§^
HOMA‐β	90 (64–113)	83 (62–108)	.3
OGTT fasting p‐glucose (mmol/l)	5.3 (0.4)	5.4 (0.4)	.5
OGTT 30 min p‐glucose (mmol/l)	8.2 (1.6)	7.9 (1.3)	.4
OGTT 120 min p‐glucose (mmol/l)	6.5 (1.3)	6.2 (1.3)	.3
OGTT fasting p‐insulin (pmol/l)	52 (43–66)	54 (41–70)	1.0^§^
OGTT 30 min p‐insulin (pmol/L)	250 (207–383)	330 (236–483)	.2
OGTT 120 min p‐insulin (pmol/L)	198 (154–300)	218 (144–316)	.8^§^
Baseline data (both offspring born to women with type 1 diabetes and matched controls)			
Gestational age at delivery (days)	264 (247–279)	268 (259–280)	.4
Birthweight (g)	3559 (734)	3558 (666)	1.0
Birthweight z‐score	1.17 (2.1)	0.82 (1.4)	.7
Section, *n* (%)	6 (40)	164 (37)	.8^†^
Maternal BMI [*n*]	22.9 (20.5–23.5) [14]	22.8 (21.0–25.1) [380]	.3
Baseline data, type 1 diabetes mothers only			
Pre‐gestational HbA_1c_, mmol/mol [%] (*n* = 226)	68 (50–90) [8.4 (6.7–10.4)]	58 (25–107) [7.5 (4.4–11.9)]	.03
1. trimester HbA_1c_, mmol/mol [%] (*n* = 246)	64 (40–76) [8.0 (5.8–9.1)]	55 (20–89) [7.2 (4.0–10.3)]	.2
2. trimester HbA_1c_, mmol/mol [%] (*n* = 253)	53 (27–61) [7.0 (4.6–7.7)]	49 (27–91) [6.6 (4.6–10.5)]	.8
3. trimester HbA_1c_, mmol/mol [%] (*n* = 247)	52 (34–66) [6.9 (5.3–8.2)]	50 (29–92) [6.7 (4.8–10.6)]	.8

Note

Data are presented as mean and standard deviation (SD) if normally distributed, and as median and interquartile range or range if skewed distributed. Type 1 diabetes exposure status, male sex, GAD65aab status and section are presented as number (*n*) and per cent (%). BMI, Body Mass Index; IFG, impaired fasting glucose; IGT, impaired glucose tolerance. Abnormal glucose tolerance = Diabetes + IFG + IGT. P‐values are generated using Student's t test or ^†^Chi2, ^‡^Fishers exact test or ^§^Wilcoxon rank‐sum test.

### 3.1 GAD65aab and abnormal glucose tolerance

One participant was diagnosed with GAD65aab‐negative diabetes during the study day, eighteen were diagnosed with IFG, and 44 with IGT (Table 1). Overall, more were GAD65aab‐positive if diagnosed with abnormal glucose tolerance (*p *= .03). There was no overall difference in GAD65aab status if the participants were diagnosed with either IFG or IGT.

### 3.2 GAD65aab, abnormal glucose tolerance and T1D exposure

We observed more offspring born to mothers with T1D being GAD65aab‐positive if diagnosed with abnormal glucose tolerance (4/36 vs. 7/220) or isolated IFG (2/9 vs. 9/247), but the difference did not reach the level of statistical significance (*p *= .07 and *p *= .07) and the difference was not present among the controls either. When looking at isolated IGT, we found no difference in GAD65aab status in any of the groups (data not shown).

### 3.3 GAD65aab and offspring or maternal characteristics

We found no association between GAD65aab status and glucose or insulin levels during OGTT, HOMA‐IR, HOMA‐β, birthweight, birthweight z‐scores, mode of delivery, current weight, weight SDS, height, height SDS, BMI, BMI‐SDS or maternal BMI prior to pregnancy (Table 1). For offspring born to women with T1D, we found maternal prepregnancy HbA_1c_ to be higher in the GAD65aab‐positive offspring when compared to GAD65aab‐negative offspring. Otherwise, we found no difference in neither offspring nor maternal characteristics between the two GAD65aab groups.

## 4 DISCUSSION

In this study, we show that among those diagnosed with pre‐diabetes more were GAD65aab‐positive. In EPICOM, we reported offspring born to women with T1D to have an increased risk of pre‐diabetes, higher BMI, reduced insulin sensitivity and relative insulin secretion deficiency.^2^ With the results of this present study, we cannot rule out that some of the cases with pre‐diabetes could be due to pre‐clinical T1D. No difference in presence of GAD65aab was found between offspring born to mother with T1D and controls. However, our study was not powered to detect this difference.

We found the frequency of GAD65aab in offspring born to women with T1D to be slightly higher, than the 3.2% that has previously been described by Bonifacio et al.^4^ The frequency of GAD65aab‐positive subjects among our controls is similar to results from two previous studies, one including adults (age 20–90 years, *n* = 4.496) and one including schoolchildren (age 6–17 years, *n* = 14.742).^11^, ^12^

We found maternal prepregnancy HbA_1c_ to be higher among the GAD65aab‐positive children. Bonifacio et al. did not report maternal prepregnancy glycaemic control, but they found a mildly elevated third‐trimester HbA_1c_ (5.7–7%) to be associated with a reduced risk of autoimmunity in the offspring, while maternal third‐trimester HbA_1c_ >7% was associated with an increased risk of autoimmunity among the offspring.^4^ They also describe offspring birth weight to be associated with the presence of islet autoantibodies in an inverted U‐shaped curve relationship (low and high birth weight being protective for T1D development).^4^ Our results do not support these findings, nor when dividing birthweight z‐scores into tertiles. It has been debated whether delivery by caesarean section is associated with an increased risk of T1D among the offspring.^13^, ^14^ In our study, we found no difference in the frequency of caesarean section between neither the GAD65aab‐positive and the GAD65aab‐negative offspring nor among the participants with abnormal glucose tolerance vs. the normoglycaemic.

An obvious limitation of our study is the lack of information on paternal and sibling T1D status. Also, the number of participants being GAD65aab‐positive was low (*n* = 17) which increases the risk of a type 2 error. However, the EPICOM study was not designed nor powered to detect any difference in the presence of GAD65aab between offspring born to women with T1D and their matched controls. This present study would have benefitted from measurements of additional islet autoantibodies associated with the development of T1D to fully describe any autoimmune background in the development of diabetes among offspring born to women with T1D. The inclusion of additional autoantibodies would also have been beneficial in predicting risk of future T1D development.^5^ However, we chose to examine GAD65aab only as it is easily accessible in Denmark and because of the age dynamics of appearance of islet antibodies, where appearance of GAD65aab best matches the age of the participants in EPICOM.^6^ Also, a recent systematic review has highlighted an association between not only GAD65aab and T1D but also GAD65aab and the development of type 2 diabetes.^15^ As offspring born to women with T1D are at higher risk of developing diabetes, the finding of screening tool for offspring at risk is relevant and the stability of GAD65aab autoantibodies over years, relative low price and accessibility of the analyses, makes it, in our opinion, a good candidate.^6^, ^16^

## 5 FUNDING STATEMENT

This study was supported by the European Foundation for the Study of Diabetes/Lilly 2015 Programme and the AP Møller Foundation. The funding sources were not involved in the collection, analysis or interpretation of data or in writing and submission of the manuscript.

## CONFLICTS OF INTEREST DISCLOSURE

SK, MCL, ZL, BB, TDC and KH declare that there is no conflict of interest associated with their contribution to this manuscript. PD and DMJ are participating in multi‐centre and multi‐national clinical studies on the use of insulin in pregnant women with pre‐existing diabetes in collaboration with Novo Nordisk, and no personal honorarium is involved. DMJ has received lecture fees from Eli Lilly and Sanofi. RBJ and CHG have received lecture fees from Novo Nordisk.

## AUTHOR CONTRIBUTION

**Sine Knorr:** Conceptualization (lead); Data curation (lead); Formal analysis (lead); Funding acquisition (lead); Investigation (lead); Methodology (lead); Project administration (lead). **Magnus C Lydolph:** Formal analysis (supporting); Methodology (supporting). **Birgitte Bytoft:** Investigation (equal). **Zuzana Lohse:** Investigation (equal). **Tine D Clausen:** Conceptualization (supporting); Funding acquisition (supporting). **Rikke Beck Jensen:** Conceptualization (supporting). **Peter Damm:** Conceptualization (supporting); Supervision (supporting); Writing‐review & editing (supporting). **Kurt Hoejlund:** Conceptualization (supporting); Supervision (supporting); Writing‐review & editing (supporting). **Dorthe Møller Jensen:** Conceptualization (supporting); Supervision (supporting); Writing‐review & editing (supporting). **Claus H Gravholt:** Conceptualization (supporting); Funding acquisition (supporting); Supervision (supporting); Writing‐review & editing (supporting).

## Data Availability

The data used for this study will be available for researchers who hold the relevant permits upon reasonable request.
